# Supplementary material for: Patterns of Intron Gain and Loss in Fungi
Source: PLoS Biol. 2004 Nov 30;2(12):e422. doi: 10.1371/journal.pbio.0020422 (PMC532390; doi:10.1371/journal.pbio.0020422)
Supplement: Table S1 — Also available at http://genes.mit.edu/NielsenEtAl/. (4.3 MB ZIP). [file pbio.0020422.st001.zip › NielsenEtAl/html/1303.html]

AN4997.1.NCU02263.1.MG00905.1.FG10779.1


```
 CLUSTAL W (1.82) Multiple Sequence Alignments - Introns Inserted


Sequence 1: NCU02263.1	334 aa
Sequence 2: MG00905.1	343 aa
Sequence 3: FG10779.1	337 aa
Sequence 4: AN4997.1	327 aa
Alignment Length: 346 aa
Number Identitical Residues: 170 aa
Alignment Score (without introns) 8685


MG00905.1 	MAAPAATAPLQLDPKYDGYDFPTVAPVPAPGHPGHLTAEQQAQVSQLRLMLESQGYTDRL
NCU02263.1	---------MELEPKYDDYDYPTTAPDAQEGHPGHLTPQQQAQVTQLRLMLESEGYTKRL
FG10779.1 	---------MASNPKYDNYDFPAQGAEKQDGHAGYLTEDQTAKLEQFRMMLESEGCTDRL
AN4997.1  	----MAAPAEKQSSKYDDYDFPTTAPEAQPGHPGHTTPEQDAKVEQLRSELEQEGYTERL
          	     ::..   ..***.**:*: ..    **.*: * :* *:: *:*  **.:* *.**

MG00905.1 	DTLTL0LRFLRARKFDVNLALKM2FVDCEKWRKETKLDEILPTWDYPEKAEIFKYYPQYY
NCU02263.1	DTLTL0LRFLRARKFDVNLAKQM2FVECEKWRAETKLDETIPEWEYPEKEEVSKYYPQYY
FG10779.1 	DTLTL0LRFLRARKFDVEASKAM2FLDTEKWRKETKLDETVPVWDYPEKAEINKYYTQFY
AN4997.1  	DTLTL~LRFLRARKFDVAAAKAM2FIASEKWRKEFGTDDLARTFEYTEKPEVFKYYPQYY
          	***** ***********  :  * *:  **** *   *:    ::*.** *: ***.*:*

MG00905.1 	HKTDK0DGRPVYIEQLGNADITAMNKITTQERMLTNLAVEYERVADPRLPACSRKSGHLL
NCU02263.1	HKTDK0DGRPVYIEQLGKIDLNAMYKITTAERMLTNLAVEYERLADPRLPACSRKAGVLL
FG10779.1 	HKTDK0DGRPIYIETLGGIDLNAMYKITTAERMLTNLAVEYERVADPRLPACSRKAGHLL
AN4997.1  	HKTDK0DGRPVYIEKLGNINIAELQKITTDERMLKNLVTEYEKLADPRLPACSRKAGKLL
          	***** ****:*** **  ::  : **** ****.**..***::***********:* **

MG00905.1 	ETCCTIMDFKGVGISKASQVYGYVRAASNMSQNYYPERLGRLYLINTPWGFSGVWGIVKG
NCU02263.1	ETCCTIMDLKGVGLTKAPQVFGYVKQASTLSQNYYPERLGKLYLINAPWGFSTVWNVIKA
FG10779.1 	ETCCTVMDLKGVSIGKVPQVYSYVKQASVISQNYYPERLGKLYMINAPWGFSTVWSIVKG
AN4997.1  	ETCCSIIDLKGVGITSAPSVYGYLKMTSAVSQNYYPERLGKLYLINAPWGFSTVFSVVKS
          	****:::*:***.: ....*:.*:: :* :**********:**:**:***** *:.::*.

MG00905.1 	WLDPVTVQKIHILGSGYQKELLAQIPAENLPKSLGGTCTCAGGCELSDAGPWNEKEWARP
NCU02263.1	WLDPVTVSKIHVLGSGYSKELLGQVPPENLPKEFGGTCQCAGGCHMSDMGPWREEQWARP
FG10779.1 	WLDPVTVSKINILGSGYKGELLKQIPAENLPKAFGGECQCEGGCENSDAGPWHEAEFARP
AN4997.1  	FLDPVTVNKIHVLGSGYQSELLKQVPKENLPQQYGGTCQCEGGCEYSDMGPWREPEWARE
          	:******.**::*****. *** *:* ****:  ** * * ***. ** ***.* ::** 

MG00905.1 	PKWETKHKQQAAAP---AAAAAPAATEPAPAAAPAAPAAEAEAKQPAAA
NCU02263.1	AKWEKKADAPAAAP---AAAPAATEAAPAPAAEPAVAATEATAPAPAAA
FG10779.1 	AWWEKKQDANVIENKGSEIEEPAKAPEAAPVAEGADATQTAPAPAPAAA
AN4997.1  	PKWANPKGNKGDVP------------AENPVAE--KPTEAPEAPQPAA-
          	. * .                        *.*    .:  . *  ***
```
